# Report on Sweating Sickness

**Published:** 1851-11

**Authors:** 


*Report on Sweating Sickness.*—M. J. Guerin, in the name of a commission composed of MM. Dubois, Melier, Martin-Solon, Bricheteau, and and Guerin, read an elaborate report on a series of documents transmitted to the Academy respecting the epidemic sweating sickness of 1849. After some general observations upon epidemics, the reporters discussed the facts and inferences contained in these documents by propounding a series of questions.

1st. Does the *sweating sickness* of 1849 resemble preceding epidemics? After reviewing the history of this disease, from the scattered and imperfect notices thereof in the writings of Galen and of Cælius Aurelianus, down to the first unequivocal account of the disease in 1465, the reporters compare the English sweating sickness with the same malady as it appeared in Picardy in 1655, and then with the epidemic that has at different times made its appearance in various parts of France, and last in the year 1849. The result of this comparative examination is, that the epidemic of 1849 is but a milder form of the English sweating sickness of the middle ages, and is also identical with the several epidemics that have occurred in France since the year 1712.

2d. Has the epidemic of 1849 reappeared in districts that it had formerly visited? Has it spared some places in which it had previously occurred, and then after a time reappeared? On these points data are yet wanting.

3d. Has the epidemic of 1849 manifested characters similar to those of its predecessors? The answer to this question demands data whereon to determine whether, although fundamentally identical with preceding epidemics, this of 1849 has presented the same forms as those: whether, in the course of the disease, it has assumed different forms; whether it has presented the same form in the different provinces, or in different places of the same province; lastly, whether the same epidemic, during the same period, and in the same localities, has presented sufficiently distinct characters to have constituted distinct types. None of the reports that have been received furnish the necessary information upon these points, although up to a certain point they supply data. The results of treatment would in a great measure assist in the resolution of this question. Here, however, the diversity and even opposition of opinions leave the inference that nervous systems have predominated, and have varied with local or originating causes. The course of this epidemic, like that of cholera, was marked by a diminished severity in the attacks towards its decline; so that from being at first severe and fatal, they became slight and unattended with danger. The diversity of opinion on the subjects of infection and of treatment, and the varieties observed in the symptoms described, present the disease under different forms, but do not supply sufficiently accurate data for the determination of the distinctness of type in different localities.

4th. Have the reports now submitted to the commission added anything to the characteristics of the sweating sickness? In considering this point the reporters confine their observations principally to the subject of the mode of propagation of the malady. It does not appear that the documents hitherto received afford the determination of this point. Some observers affirm absolutely its contagiousness; others explicitly deny this property. One physician states that those only who had long resided in the district where the disease appeared were liable to its attacks; that strangers were exempt. This fact, the reporters observe, is analogous to what is recorded by historians of the disease in Calais, whither *it was imported by the English*, and where it was confined to the English inhabitants; whence its name of English sweating sickness.

5th. What is the nature of the epidemic of 1849? Some of the authors speak of a change in the nature of the disease: upon this the commissioners observe that the nature of the disease must remain unaltered, although its manifestations may be modified by circumstances. Opinions are divided into those which regard the disease as inflammatory, and those which regard it as a septic malady, involving at once the whole mass of circulating fluids. Of the six reports now under notice, not one speaks of the disease as inflammatory (gastritis), but unanimously look upon it as septic.

6th. What treatment has prevailed in the epidemic of 1849, and what progress has the study of the therapeutic relations of the disease impressed upon its history? The treatment of sweating sickness is far from fixed; bleeding, antiperiodics, and evacuants, have all been used with varying success. Some have vaunted the success of depletion, while others have deplored the fatal effects of that measure; some have cured their patients with quinine, others by the employment of emetics. These opposing statements are only reconcilable by assuming variations in the period or characters of the epidemic. One report speaks of six hundred cases cured by bleeding; another records the cure of one thousand by emetics of ipecacuanha; a third gives uniform success to the combination of quinine with purgatives. The majority of cures have been obtained in various hands by emetics; but the commissioners observe that it cannot yet be asserted that ipecacuanha is the specific remedy for sweating sickness.—*London Medical Gazette.*

